# An Integrated Approach for the Comprehensive Characterization of Metabolites in Broccoli (*Brassica oleracea* var. *Italica*) by Liquid Chromatography High-Resolution Tandem Mass Spectrometry

**DOI:** 10.3390/plants14203223

**Published:** 2025-10-20

**Authors:** Zhiwei Hu, Meijia Yan, Chenxue Song, Muneo Sato, Shiwen Su, Sue Lin, Junliang Li, Huixi Zou, Zheng Tang, Masami Yokota Hirai, Xiufeng Yan

**Affiliations:** 1 National and Local Joint Engineering Research Center of Ecological Treatment Technology for Urban Water Pollution, Zhejiang Provincial Key Laboratory of Water Ecological Environment Treatment and Resource Protection, College of Life and Environmental Science, Wenzhou University, Wenzhou 325035, China; zhiwei.hu@wzu.edu.cn (Z.H.); 22451335051@stu.wzu.edu.cn (M.Y.); 22451335033@stu.wzu.edu.cn (C.S.); suelin2017@wzu.edu.cn (S.L.); lijunliang@wzu.edu.cn (J.L.); zjuzhx@wzu.edu.cn (H.Z.); 2 RIKEN Center for Sustainable Resource Science, Yokohama 230-0045, Japan; muneo.sato@riken.jp; 3 Southern Zhejiang Key Laboratory of Crop Breeding, Wenzhou Academy of Agricultural Sciences, Wenzhou Vocational College of Science and Technology, Wenzhou 325006, China; sushiwen@wzvcst.edu.cn

**Keywords:** broccoli floret, UHPLC-QTOF MS/MS, orthogonal chromatographic separation, phytochemical profiling, database

## Abstract

Background: Broccoli contains diverse phytochemicals, including glucosinolates and their hydrolysis products, with potential nutritional and bioactive properties. Accurate metabolite profiling requires optimized sample preparation and comprehensive databases. Methods: A rapid enzymatic deactivation method with 70% methanol, implemented prior to cryogrinding, was evaluated for processing freeze-dried and fresh broccoli florets, which were compared as plant materials. A widely targeted, organ-resolved metabolite database was constructed by integrating over 612 reported phytochemicals with glucosinolate degradation products. LC-HRMS combined with MS-DIAL and GNPS was employed for metabolite detection and annotation. Results: Freeze-dried samples yielded nearly twice the number of glucosinolates, isothiocyanates, and nitriles compared with standard-processed fresh tissue. Methanol pre-treatment preserved metabolite integrity in fresh samples, achieving comparable sensitivity to freeze-dried material. Using the integrated database, 998 metabolites were identified or tentatively characterized, including amino acids, carboxylic acids, phenolics, alkaloids, terpenoids, and glucosinolate derivatives. Cross-platform reproducibility was improved and false positives reduced. Conclusions: Optimized sample preparation combined with a curated metabolite database enables high-confidence, comprehensive profiling of broccoli florets phytochemicals. The resulting dataset provides a valuable reference for studies on genotype–environment interactions, nutritional quality, and functional bioactivity of cruciferous vegetables.

## 1. Introduction

Comprehensive identification of plant metabolites is essential for developing, managing, and utilizing food composition databases, which serve as valuable resources for nutrition, health, breeding, and food processing applications [1,2,3]. However, the extensive structural diversity, wide concentration ranges, and limited availability of analytical standards pose significant challenges for fully characterizing food metabolomes. This bottleneck directly impacts the completeness, accuracy, and utility of food composition databases, which rely on extensive, high-resolution metabolic profiles to aid in diet formulation, health policy, and agricultural innovation.

Sample storage is a critical first step in metabolomic studies, as preservation methods directly affect the stability and detectability of metabolites. Freeze-drying is widely regarded as one of the most effective techniques for plant-based food preservation, since it removes water under low temperature and vacuum, thereby minimizing thermal and enzymatic degradation. Its key advantages include excellent retention of nutritional quality, bioactive compounds. Freeze-drying also stabilizes labile metabolites, making samples highly suitable for metabolomic analyses and long-term storage. However, the technique has notable drawbacks: it is time- and energy-intensive, requires specialized equipment, and involves long processing cycles. Despite these limitations, freeze-drying remains the benchmark method for preserving plant-based foods when the goal is to maximize retention of bioactive and nutritional quality [4,5,6].

To enable more comprehensive food metabolite profiling, liquid chromatography-mass spectrometry (LC-MS) methods have become indispensable, and ongoing advancements in data acquisition, processing, and interpretation are helping maximize coverage and confidence in metabolite annotations [7,8]. The implementation of high-resolution tandem mass spectrometry (HR-MS/MS) platforms, such as Q-TOF, Q-Orbitrap, and Triple-TOF, further improves both the depth and accuracy of identifications [9,10]. Nonetheless, co-eluting compounds and overlapping signals can undermine coverage, particularly for low-abundance compounds. To address these issues, advanced orthogonal-phase liquid chromatography methods—combining normal-phase hydrophilic interaction (HILIC) with reverse-phase C18—can aid in their resolution and enable more exhaustive metabolic profiles [11,12].

Post-acquisition data processing and interpretation remain bottlenecks for the large-scale characterization of food metabolomes [13,14]. Targeted matching against compound databases and the use of predictive tools (e.g., Waters UNIFI, MassHunter, Thermo Compound Discoverer) streamline the identification of known compounds but are limited by the completeness of available references and the complexity of the data [15,16,17]. Open-source platforms, such as MS-DIAL [18] and Global Natural Products Social Molecular Networking (GNPS) [19], enable substructure-specific and network-informed annotations, thereby strengthening identification and reducing redundancy. Integrating these strategies with molecular networking holds significant promise for improving identification accuracy, expanding coverage, and ultimately strengthening food composition databases as a tool for health and policy decisions.

Broccoli (*Brassica oleracea* var. *italica*), a biennial cruciferous vegetable, exemplifies the growing need for a comprehensive food composition database. Global production and consumption of broccoli have surged in recent decades [20], reflecting growing awareness of its rich nutritional profile and health-promoting properties [21]. Broccoli contains a wide array of bioactive compounds, including glucosinolates (GSLs), polyphenols, flavonoids, vitamins, and dietary fiber [22], with growing evidence linking their intake to reduced disease risk [23,24]. Previous broccoli metabolomics studies have been constrained by methodological and reference-library limitations, with only 612 unique phytochemicals reported across organs, far fewer than expected for this species (Table S1) [21,25,26,27,28,29,30,31,32,33,34,35,36,37,38,39,40].

In this study, to address these gaps, an integrated analytical workflow was developed that combines optimized extraction, orthogonal chromatographic separation, high-resolution MS/MS acquisition, and both non-targeted and targeted annotation strategies to enable comprehensive characterization of the diverse metabolites in broccoli florets. We hypothesized that optimized extraction protocols that inactivate endogenous enzymes, together with integration of a curated reference database across MS-DIAL, GNPS, and SCIEX OS platforms, would enable broader and more confident metabolite annotation compared with standard workflows. The novelty of this study lies in (i) compiling the most comprehensive broccoli metabolite inventory to date, (ii) comparing freeze-drying with methanol pre-treatment for metabolite stability, (iii) employing orthogonal chromatographic separation with dual ionization modes to maximize coverage, and (iv) ensuring reliability through cross-platform integration and orthogonal validation. This strategy yielded 998 tentatively annotated and 114 confirmed compounds, substantially expanding the known phytochemical diversity of broccoli and providing a transferable framework for high-confidence metabolomics of cruciferous vegetables and other complex plant matrices. The resulting data can be directly integrated into food composition databases, advancing their utility for health, breeding, policy, and food processing applications.

## 2. Results

### 2.1. Optimization of Sample Preparation Methods

To establish an optimized methodology for broccoli metabolite extraction and analysis, freeze-dried and fresh broccoli florets were systematically compared, and sample preparation protocols were refined. The extracts were analyzed via LC-HR MS/MS, and the methodology was rigorously evaluated using qualitative performance metrics, including targeted metabolite coverage, detection sensitivity, and molecular specificity aligned with broccoli’s phytochemical profile.

Freeze-dried matrices and 70% methanol (*v*/*v*) extraction remain the predominant methodology for broccoli metabolite studies, as evidenced by the recent literature [41,42,43]. For fresh tissue processing, standard protocols involve cryogenic grinding under liquid nitrogen followed by organic solvent extraction. Non-targeted LC-HR MS/MS analysis revealed significantly reduced metabolite recovery in fresh samples (16 GSLs, 8 isothiocyanates, 7 nitriles) compared to freeze-dried counterparts (38 GSLs, 13 isothiocyanates, 11 nitriles), suggesting potential enzymatic hydrolysis of GSLs by endogenous myrosinase during sample processing—a phenomenon previously documented [44]. To address this limitation, an enzymatic deactivation strategy was implemented by introducing pure methanol (2 mL/g fresh weight, achieving 70% final concentration) prior to cryogrinding at −20 °C. This optimized protocol effectively preserved glucosinolate integrity, demonstrating comparable hydrolysis mitigation efficiency to freeze-drying-based methods.

### 2.2. The Widely Targeted Metabolomics Database from Public Data

#### 2.2.1. A Database on Published Components in Broccoli

A custom-curated broccoli phytochemical database was systematically compiled through exhaustive literature mining, with standardized descriptors including compound nomenclature, molecular formulas, organ-specific localization (floret/leaf/stem/seed/sprout), CAS registry identifiers, and primary literature sources. Following rigorous validation, the database (Table S1) encompasses 612 unique phytochemicals, with comparative organ-specific distribution analyses revealing 442 metabolites in florets, 323 in leaves, 260 in stems, 35 in sprouts, and 21 in seeds [21,25,26,27,28,29,30,31,32,33,34,35,36,37,38,39,40]. This multidimensional annotation framework provides critical insights into broccoli’s spatial metabolic architecture.

#### 2.2.2. A Database on Published Glucosinolates and Related Compounds

To systematically elucidate GSL metabolism in broccoli, a specialized database integrating GSLs and their enzymatic degradation derivatives was developed. This predictive framework was established through systematic integration of established glucosinolate degradation pathways [45,46], enabling comprehensive annotation of hydrolysis products. LC-HR MS-driven analysis successfully identified 149 GSLs and their corresponding isothiocyanate and hydrazine hydrolysates (Table S2), providing critical insights into broccoli’s dynamic glucosinolate-myrosinase system.

#### 2.2.3. A Database on Possible Metabolites in Plants

Leveraging the published targeted metabolomics framework [47,48], multiple reaction monitoring (MRM) transitions for 415 phytochemicals were systematically optimized through empirical refinement of 61,920 spectral acquisitions from 860 authenticated reference standards. Building upon this validated methodology, a putative metabolite annotation pipeline was established within the plant database, incorporating standardized descriptors: chemical nomenclature, molecular formulas, CAS registry identifiers, and chemical compound classification (Table S3).

### 2.3. Comprehensive Characterization of the Metabolites from Broccoli

**Figure 1:**
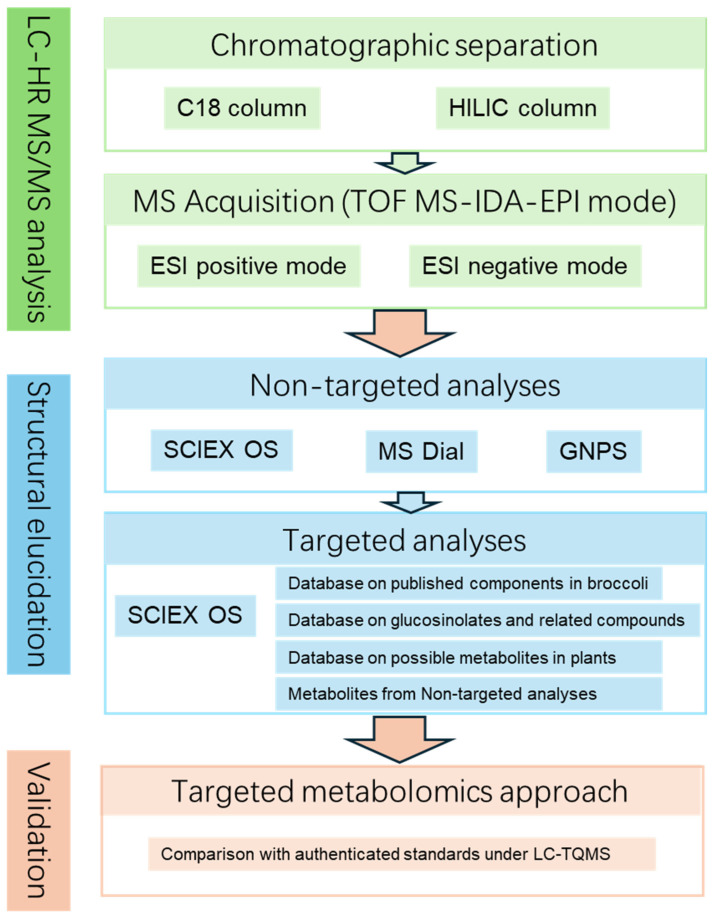
A workflow of the integrated approach for global profiling of multi-type constituents.

Comprehensive phytochemical profiling of broccoli was conducted through dual-column chromatography (C18 and HILIC columns) coupled with HR MS/MS in both positive and negative ESI modes. Full-scan MS and MS/MS spectra were acquired in TOF MS-IDA-EPI mode. Data processing incorporated complementary strategies: (1) a multidimensional non-targeted screening strategy integrating SCIEX OS, MS-DIAL, and GNPS platforms; (2) targeted verification in SCIEX OS based on curated phytochemical databases, including reported broccoli components, glucosinolates and derivatives, general plant metabolites, and candidate compounds from non-targeted analyses. Annotation reliability was ensured through a multistage validation protocol involving standardized data processing, orthogonal spectral verification (EIC integrity, isotopic fidelity, and MS/MS fragment congruence), and manual curation to resolve redundancy and ambiguity; and (3) Final validation was achieved through comparison with authenticated standards by LC-TQMS, ensuring high confidence in metabolite assignments (Figure 1).

**Figure 2:**
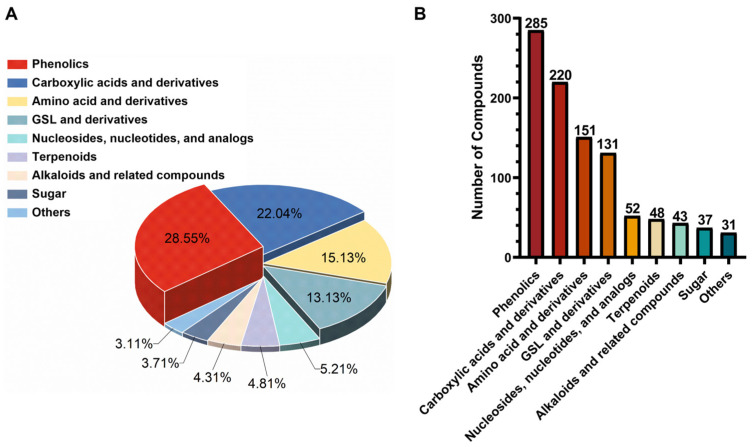
Metabolites in broccoli florets. (**A**): chemical classes distribution, (**B**): number of compounds per chemical class.

All components identified by non-targeted analyses on MS Dial and GNPS and targeted analyses were finally manually reviewed using SCIEX OS software (version 2.0) via MS and MS/MS matches. In total, 998 compounds were tentatively characterized, including 151 amino acid and derivatives, 220 carboxylic acids and derivatives, 131 GSL and derivatives, 285 phenolics, 43 alkaloids and related compounds, 52 nucleotides and analogs, 37 sugars, 48 terpenoids and 31 others (Figure 2) (Table S4). To validate the annotation accuracy of broccoli metabolites. Final validation was performed using the widely targeted metabolomics method. Through systematic comparison with authenticated reference standards analyzed under identical LC-MS/MS conditions, 114 metabolites were conclusively validated (Table 1). Here, a few representative cases were illustrated to demonstrate the employed structural elucidation approach.

**Figure 3:**
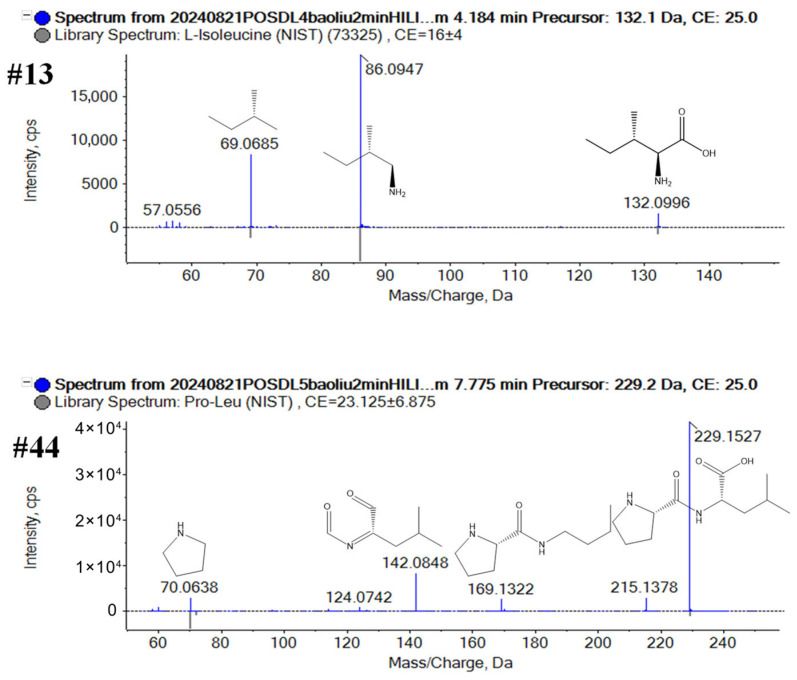

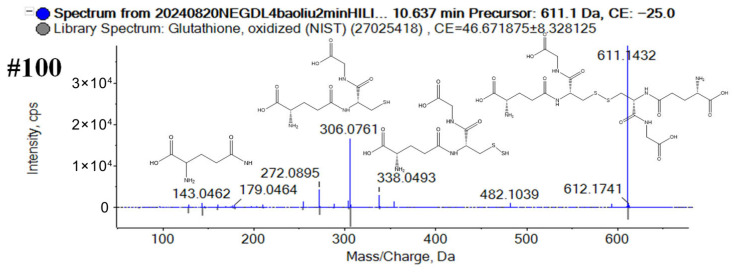
Structural elucidation of the amino acids and derivatives from broccoli by annotating the MS2 spectra of the representative compounds #13, #44, #100.

Characterization of amino acids and derivatives: A total of 151 amino acid-related compounds, including free amino acids, di/tri-peptides, and their derivatives, were structurally characterized. These polar metabolites were preferentially retained and ionized under HILIC column, consistent with their zwitterionic properties. The regular fragmentation pathways of these amino acids and derivatives involved the neutral loss of carboxylic acid moiety (CHO_2_ *m*/*z* 45.021). For exemplification, Compound #13 (t_R_ 4.1 min on the HILIC column in positive mode; *m*/*z* 132.1013 for [M+H]^+^) was identified as isoleucine (C_6_H_13_NO_2_), an amino acid. The precursor ion at *m*/*z* 132.1013 could generate the product ions of *m*/*z* 86.0947 [M+H-CH_2_O_2_]^+^ and 69.0685 [M+H-CH_2_O_2_-NH_3_]^+^. The product ion at *m*/*z* 69.0685 is typically associated with isoleucine, not leucine [49]. According to the accurate molecular weight, fragment information, and comparison with the Sciex OS library, #13 was recognized as isoleucine. Compound #44 was separated at 7.6 min on the HILIC column in positive mode, a protonated ion at *m*/*z* 229.1543 ([M+H]^+^, C_11_H_21_N_2_O_3_). In the MS^2^ spectra (Figure 3), the fragment ions were observed at *m*/*z* 215.1378 for [M+H-CH_2_]^+^, *m*/*z* 169.1322 for [M+H-CH_2_-CH_2_O_2_]^+^, *m*/*z* 142.0848 for [M+H-C_4_H_7_N-H_2_O]^+^, and *m*/*z* 70.0638 for [C_4_H_8_N]^+^. According to the accurate molecular weight and fragment information, #44 was recognized as Pro-Leu, a dipeptide compound. Compound #100 (t_R_10.9 min on the HILIC column in negative and positive modes; *m*/*z* 611.1454 for [M−H]^−^ and *m*/*z* 613.1580 for [M+H]^+^) was identified as oxidized glutathione (C_20_H_32_N_6_O_12_S_2_), oxidized form of a tripeptide compound. The deprotonated ion could generate the product ions of *m*/*z* 482.1039 ([M−H-C_5_H_7_NO_3_]^−^), *m*/*z* 338.0493 ([C_10_H_16_N_3_O_6_S_2_]^−^), *m*/*z* 306.0761 ([C_10_H_16_N_3_O_6_S]^−^ glutathione), *m*/*z* 272.0895 ([C_10_H_16_N_3_O_6_S-H_2_S]^−^), and *m*/*z* 143.0462 ([C_10_H_16_N_3_O_6_S-H_2_S-C_5_H_7_NO_3_]^−^) (Figure 3).

**Figure 4:**
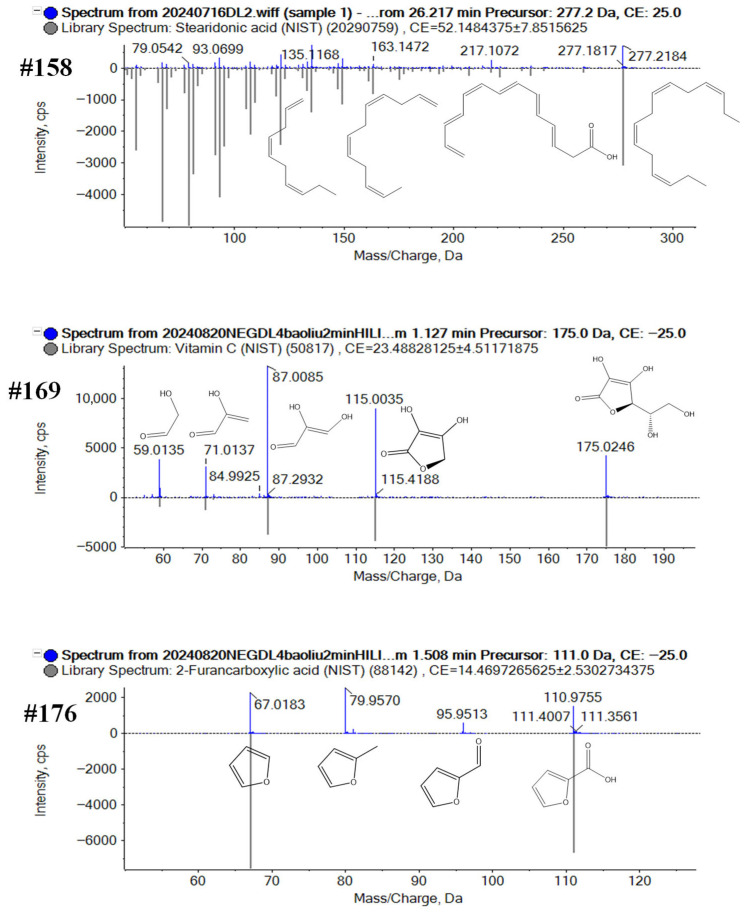
Structural elucidation of the carboxylic acids and derivatives from broccoli by annotating the MS2 spectra of the representative compounds #158, #169, #176.

Characterization of carboxylic acids and derivatives: A total of 220 compounds of these carboxylic acid compounds were characterized by both positive and negative ESI modes. Compound #158 (t_R_ 1.0 min on the HILIC column and t_R_ 26.3 min on the T3 column with [M+H]^+^ ions at *m*/*z* 277.2159 and 277.2164) was identified as stearidonic acid (C_18_H_28_O_2_). The precursor ion could generate the product ions of *m*/*z* 217.1072 ([M+H-C_2_H_4_O_2_]^+^), *m*/*z* 163.1472 ([C_12_H_19_]^+^), *m*/*z* 135.1168 ([C_10_H_15_]^+^), and *m*/*z* 93.0699 ([C_7_H_9_]^+^), *m*/*z* 79.0542 ([C_6_H_7_]^+^), which were consistent with MS^2^ spectra of stearidonic acid in Sciex OS library. Compound #169 was separated at 1.2 min on the HILIC column and 8.47 min on the T3 column with deprotonated ions at *m*/*z* 175.0252 and *m*/*z* 175.0247 ([M−H]^−^), which was identified as ascorbic acid (C_6_H_8_O_6_). In the MS^2^ spectra (Figure 4), the fragment ions were observed at *m*/*z* 115.0035 for [M−H-C_2_H_4_O_2_]^−^, *m*/*z* 87.0085 for [C_3_H_3_O_3_]^−^, at *m*/*z* 71.0137 for [C_3_H_3_O_2_]^−^, and at *m*/*z* 59.0135 for [C_2_H_3_O_2_]^−^. According to the accurate molecular weight, fragment information, and comparison with the Sciex OS library, #169 was recognized as ascorbic acid (C_6_H_8_O_6_). Compound #176 (t_R_ 1.5 min on the HILIC column in negative mode; t_R_ 2.24 min on the T3 column in negative mode; *m*/*z* 111.0089 and *m*/*z* 111.0088 for [M−H]^−^) was identified as 2-Furoic acid (C_5_H_4_O_3_). The precursor ion could generate the product ions of *m*/*z* 95.9513 ([M−H-O]^−^, *m*/*z* 79.9570 ([M−H-O_2_]^−^, and *m*/*z* 67.0183 ([C_4_H_3_O]^−^, which were consistent with MS^2^ spectra of 2-Furoic acid in Sciex OS library (Figure 4).

**Figure 5:**
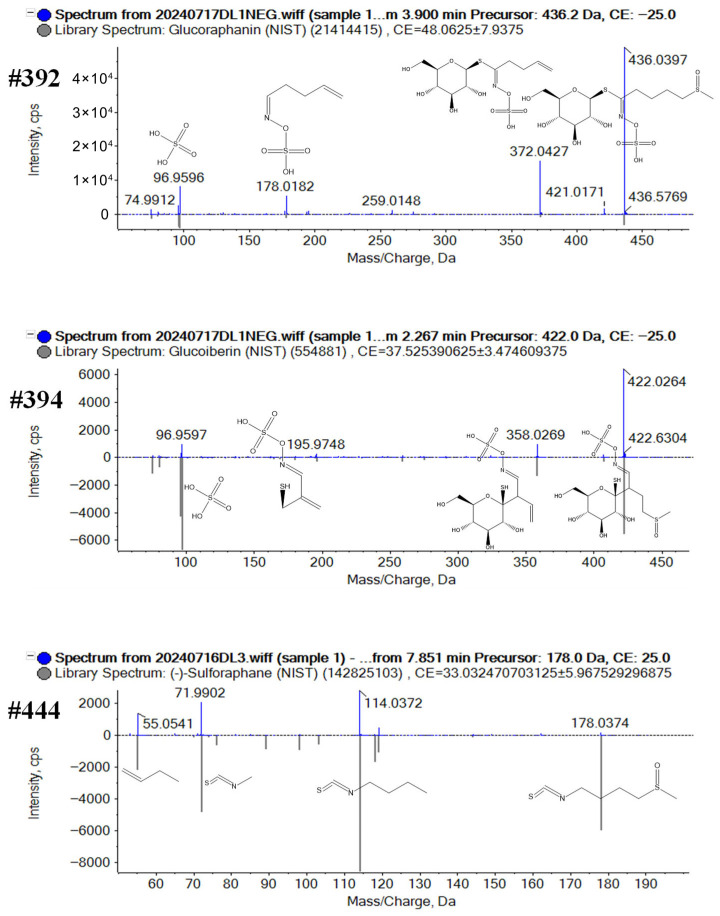
Structural elucidation of the GSL and derivatives from broccoli by annotating the MS2 spectra of the representative compounds #392, #394, #444.

Characterization of GSL and derivatives: A total of 131 compounds of this GSL-type compounds were characterized, including GSLs, isothiocyanate, and nitrile, which were identified from both positive and negative ESI mode. GSLs are more easily ionized in negative mode, the regular fragmentation pathways of these GSLs involve the neutral loss of sugar and sulfate moiety. Compound #392 was separated at 3.4 min on the HILIC column and 3.8 min from the T3 column in negative mode with deprotonated ions at *m*/*z* 436.0409 and *m*/*z* 436.0415 ([M−H]^−^, C_12_H_23_NO_10_S_3_). In the MS^2^ spectra (Figure 5), the fragment ions were observed at *m*/*z* 372.0427 for [M−H-CH_4_OS]^−^, at *m*/*z* 178.0182 for [M−H-CH_4_OS-C_6_H_11_O_5_S]^−^, and at *m*/*z* 96.9596 for [SO_4_H]^−^. According to the accurate molecular weight, fragment information, and comparison with the Sciex OS library, #392 was recognized as glucoraphanin. Compound #394 (t_R_ 3.1 min on the T3 column and 4.1 min on the HILIC column; *m*/*z* 422.0256 and *m*/*z* 422.0255 for [M−H]^−^) was identified as glucoiberin (C_11_H_21_NO_10_S_3_). The precursor ion could generate the product ions of *m*/*z* 358.0269 ([M−H-CH_4_OS]^−^), *m*/*z* 195.9748 ([C_4_H_6_NO_4_S_2_]^−^), and *m*/*z* 96.9597 ([SO_4_H]^−^). As for Isothiocyanates, it was mainly able to be ionized in positive mode. Compound #444 (t_R_ 7.9 min on the T3 column; *m*/*z* 178.0354 for [M+H]^+^) was identified as sulforaphane (C_6_H_11_NOS_2_). The precursor ion generated the product ions of *m*/*z* 114.0372 ([M+H-CH_4_OS]^+^), *m*/*z* 71.9902 ([M+H-CH_4_OS-C_3_H_6_]^+^), and *m*/*z* 55.0541 ([C_4_H_7_]^+^) (Figure 5).

**Figure 6:**
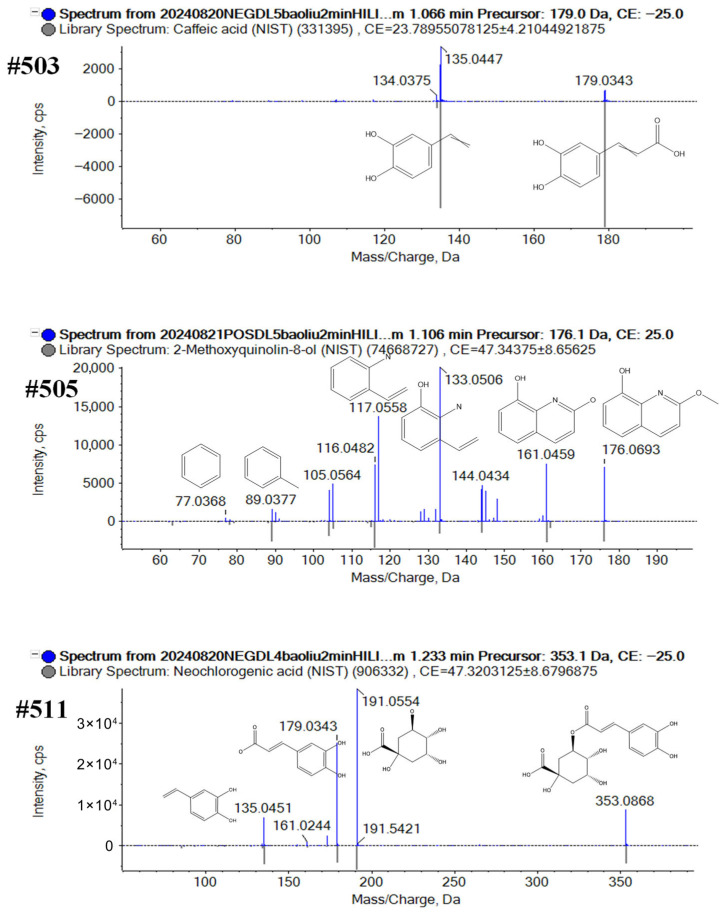
Structural elucidation of the phenolics from broccoli by annotating the MS2 spectra of the representative compounds #503, #505, #511.

Characterization of Phenolics: A total of 285 phenolic compounds were characterized by both positive and negative ESI modes. Taking compound #503 (t_R_ = 1.1 min on the HILIC column and t_R_ = 5.71 min on the T3 column in negative mode, *m*/*z* 179.0350 and *m*/*z* 179.0351 for [M−H]^−^) as an example for illustration, the molecular formula was initially deduced as C_9_H_8_O_4_ (mass error, 0.3 and 0.6 ppm). According to the MS/MS spectrum, compound #503 easily eliminated the unit of carboxylic acid to generate an abundant product ion of *m*/*z* 135.0375. According to the accurate molecular weight, fragment information, and comparison with the Sciex OS library, #503 was recognized as caffeic acid. Compound #505 (t_R_ 1.1 min on the HILIC column and t_R_ 5.7 min on the T3 column in positive mode; *m*/*z* 176.0700 and *m*/*z* 176.0707 for [M+H]^+^) was identified as 2-methoxyquinolin-8-ol (C_10_H_9_NO_2_). The precursor ion could generate the product ions of *m*/*z* 161.0459 ([M+H-CH_3_]^+^), *m*/*z* 133.00506 ([M+H-CH_3_-CO]^+^), *m*/*z* 117.0558 ([M+H-CH_3_-CO_2_]^+^), *m*/*z* 89.0377 ([C_6_H_3_CH_3_+H]^+^), and *m*/*z* 77.0368 ([C_6_H_4_+H]^+^), which were consistent with MS^2^ spectra of 2-methoxyquinolin-8-ol in Sciex OS library. Compound #511 was separated at 8.9 min on the T3 column in negative mode with a deprotonated ion at *m*/*z* 353.0881 ([M−H]^−^, C_16_H_18_O_9_). In the MS^2^ spectra, the fragment ions were observed at *m*/*z* 191.0554 for [C_7_H_11_O_6_]^−^ (corresponding to quinic acid), *m*/*z* 179.0343 for [M−H-C_7_H_11_O_5_]^−^, and at *m*/*z* 135.0451 for [M−H-C_7_H_11_O_5-_CO_2_]^−^. According to the accurate molecular weight, fragment information, and comparison with the Sciex OS library, #511 was recognized as neochlorogenic acid (Figure 6).

## 3. Discussion

### 3.1. Optimization of Sample Preparation Methods

Our comparison of freeze-dried and fresh broccoli matrices highlights how strongly sample preparation influences the reliability of LC-HRMS metabolomics data. Freeze-drying followed by 70% methanol extraction remains the benchmark in broccoli metabolite studies because it minimizes enzymatic activity, improves extraction efficiency, and yields broader metabolite coverage [24,44]. In our study, freeze-dried material produced nearly twice as many glucosinolates, isothiocyanates, and nitriles as fresh tissue processed under standard cryogenic grinding protocols. This difference mirrors previous reports attributing diminished recovery in fresh tissue to residual myrosinase activity during handling [44]. Importantly, the introduction of a rapid enzymatic deactivation step—adding pure methanol to reach a 70% final concentration before cryogrinding at −20 °C—substantially mitigated the loss of key metabolites in fresh samples. This approach not only preserved glucosinolate integrity but also achieved sensitivity and specificity comparable to freeze-dried material, making it a practical alternative for laboratories that need to process fresh material quickly or lack freeze-drying capacity. Overall, our findings reinforce that method optimization at the sample-preparation stage is essential for accurately characterizing the complex phytochemical composition of broccoli and for reducing biological and technical variability in downstream analyses.

### 3.2. Orthogonal Chromatographic Separation

**Figure 7:**
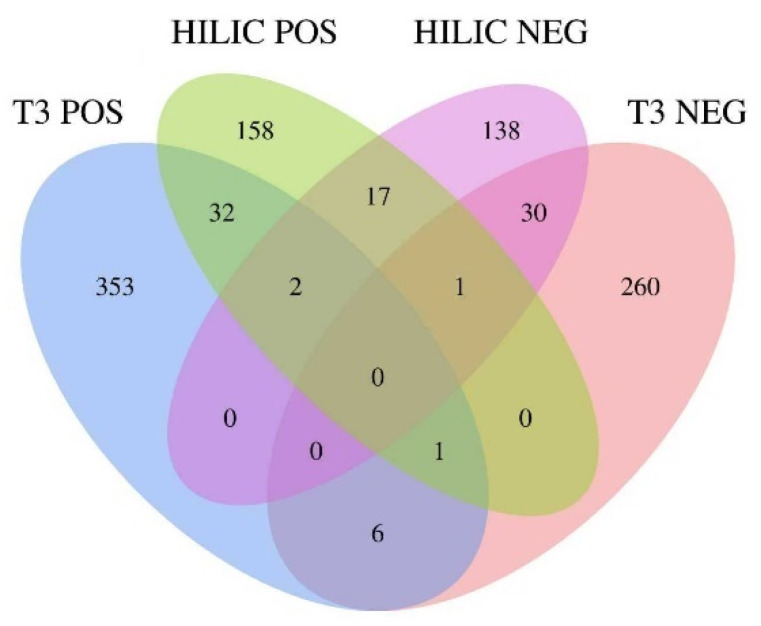
Venn diagram showing metabolite identifications across four LC–MS/MS modes: T3 POS, T3 NEG, HILIC POS, and HILIC NEG.

The Venn diagram (Figure 7) illustrates the distribution and overlap of metabolites detected across four LC–MS/MS acquisition modes: T3 positive (T3 POS), T3 negative (T3 NEG), HILIC positive (HILIC POS), and HILIC negative (HILIC NEG). A large proportion of metabolites were identified uniquely in single modes, such as 353 in T3 POS, 260 in T3 NEG, 158 in HILIC POS, and 138 in HILIC NEG, underscoring the distinct selectivity of each chromatographic–ionization condition. In contrast, only small subsets of compounds were shared between two or more modes, such as 32 between T3 POS and HILIC POS, 30 between HILIC NEG and T3 NEG, and 17 between HILIC POS and HILIC NEG. Notably, only a very limited number of metabolites were consistently detected across three or four modes, reflecting the chemical diversity and ionization specificity inherent in plant metabolomes.

These findings highlight the strength of employing orthogonal chromatographic separation combined with dual-polarity acquisition. The reversed-phase T3 column favors retention of moderately polar and hydrophobic metabolites, while the HILIC column enhances the coverage of highly polar and ionic compounds. Similarly, positive and negative ESI modes provide complementary ionization efficiency for different chemical classes, such as amines, alkaloids, organic acids, and glucosinolates. By integrating both chromatographic dimensions and ionization polarities, we achieved markedly broader metabolite coverage than would be possible with any single analytical condition. This multidimensional approach reduces the risk of overlooking metabolites restricted to a specific chemical or ionization niche and thereby enhances both the sensitivity and comprehensiveness of the profiling workflow.

### 3.3. Integration of Widely Targeted Metabolomics Databases

Developing a comprehensive, publicly traceable phytochemical database for broccoli allowed us to contextualize and validate our LC-HRMS data across multiple compound classes. By integrating published records for 612 phytochemicals and expanding coverage of glucosinolates and their hydrolysis products [20,45], an organ-resolved reference was created that mirrors the spatial metabolic organization of broccoli tissue. This database served as a foundation for aligning non-targeted and targeted analyses and for prioritizing compounds that might otherwise escape detection. In particular, incorporating glucosinolate degradation pathways and putative plant metabolites enabled more confident annotation of both known and novel compounds, bridging gaps left by conventional spectral libraries [20,48]. When combined with our multidimensional analytical workflow (SCIEX OS, MS-DIAL, and GNPS), the curated database greatly increased annotation accuracy, reduced false positives, and improved cross-platform reproducibility. This synergistic approach illustrates the value of integrating literature-derived knowledge with high-resolution MS/MS data for expanding the chemical space detectable in plant metabolomics. Ultimately, the merged database–analytical pipeline enabled us to identify and/or tentatively characterize 998 compounds in broccoli, including low-abundance metabolites across amino acids, carboxylic acids, phenolics, terpenoids, and glucosinolate derivatives. Such comprehensive coverage provides a new baseline for future work on genotype–environment interactions, nutritional quality, and functional bioactivity of cruciferous vegetables.

### 3.4. Linking Methods to Comprehensive Metabolite Characterization

Combining optimized sample preparation with a richly annotated database enabled the most comprehensive profiling of broccoli metabolites to date. Stabilizing labile compounds during extraction and aligning high-resolution MS/MS outputs with organ-specific references yielded unprecedented breadth and depth, including low-abundance amino acids, carboxylic acids, phenolics, terpenoids, and multiple glucosinolate derivatives. This dual strategy supported both non-targeted discovery and targeted verification, facilitating cross-platform consistency and stringent false-discovery control. The approach outlined here establishes a methodological framework for future studies on genotype–environment interactions, nutritional quality, and bioactivity of cruciferous vegetables. It also sets the stage for the in-depth structural elucidation presented in Section 2.3, where our combined workflows converge to achieve confident characterization of over a thousand broccoli metabolites.

### 3.5. Summary of Comprehensive Metabolite Characterization

Through our integrated non-targeted and targeted LC-HRMS workflows, a total of 998 metabolites were tentatively characterized, spanning amino acids and derivatives, carboxylic acids and derivatives; glucosinolates and derivatives; phenolics; alkaloids and related compounds; terpenoids; nucleosides, nucleotides and analogs; sugars; and other compound classes. This represents more than twofold increase compared with the 442 metabolites previously reported in broccoli floret. Glucosinolates and derivatives are a class of characterized metabolites in broccoli, but most earlier metabolomic studies identified only 22 glucosinolates (Table S1) in total and focused primarily on 8–15 glucosinolates [50,51,52,53]. By applying dual chromatographic separation and cross-platform annotation, our workflow enabled the identification of 131 glucosinolates and derivatives, including 78 distinct glucosinolates and their downstream derivatives, 32 isothiocyanates and 21 nitriles, thereby substantially expanding the known phytochemical diversity of broccoli. Orthogonal verification with authenticated standards further confirmed 114 key metabolites, underscoring the reliability and high confidence of the annotations. Collectively, this extensive inventory highlights the remarkable chemical diversity but also establishes a high-quality benchmark reference that can support future studies on functional activity, nutritional value, biomarker discovery, and targeted breeding of health-promoting *Brassica* cultivars.

### 3.6. Physiological, Nutritional, and Pharmacological Significance

The expanded inventory of broccoli metabolites identified in this study carries important physiological, nutritional, and pharmacological implications. Many of the compounds characterized—especially glucosinolates and phenolics—are integral to plant defense mechanisms and stress responses, offering insight into the biochemical basis of broccoli’s resilience and adaptive traits. Nutritionally, the organ-resolved metabolite profiles reveal distinct distributions of amino acids, vitamins, phenolics, and terpenoids, directly linking phytochemical composition with the health-promoting properties of broccoli as a dietary component. Pharmacologically, the detection of bioactive metabolites such as sulforaphane precursors, indole derivatives, and antioxidant flavonoids expands the evidence base connecting broccoli consumption with reduced risks of cancer, cardiovascular disease, and metabolic disorders.

In addition, the functional implications of metabolite distribution provide a deeper understanding of broccoli metabolism and stress adaptation. Phenolics with strong antioxidant properties, enriched particularly in leaves, likely contribute to photoprotection and redox homeostasis under high light or abiotic stress while also enhancing the antioxidant capacity of broccoli in the human diet. Glucosinolates, which accumulated predominantly in florets and young tissues, serve as critical defense metabolites and precursors of bioactive hydrolysis products such as isothiocyanates and nitriles. Their abundance underscores both ecological functions against herbivores and pathogens and nutritional significance in cancer chemoprevention.

## 4. Materials and Methods

### 4.1. Chemicals and Reagents

Acetonitrile (LC grade, Cat. No. 34851-4X4L, Sigma-Aldrich, St. Louis, MO, USA); methanol (LC grade, Cat. No. 34860-4X4L-R, Sigma-Aldrich, St. Louis, MO, USA); formic acid (LC-MS grade, Cat. No. 5330020050, Sigma-Aldrich, St. Louis, MO, USA); ultrapure water (Smart2Pure, Thermo Scientific, Waltham, MA, USA). All other chemicals were of analytical reagent grade and were purchased from commercial suppliers.

### 4.2. Plant Materials and Sample Preparation

Seeds of six broccoli cultivars were obtained directly from the original commercial suppliers to ensure varietal purity and genetic authenticity, which were formally confirmed by Shiwen Su and Zheng Tang (Wenzhou Vocational College of Science and Technology) (Table 2). All cultivars were cultivated under standardized management practices at the experimental base of the Wenzhou Academy of Agricultural Sciences, Zhejiang, China (120.51° E, 28.06° N; elevation 10 m). Florets were harvested at the commercial maturity stage during December 2022 and January 2023, immediately frozen in liquid nitrogen, and subsequently stored at −80 °C prior to freeze-drying. To obtain more comprehensive coverage of metabolites and improve detection efficiency, a pooled sample strategy was employed. Approximately 5 g of florets from each cultivar were freeze-dried using a vacuum freeze dryer (LGJ-12, Songyuan Huaxing Technology Development Co., Ltd., Beijing, China) at a condenser temperature of −60 °C and a pressure of 5 Pa for 48 h. The freeze-dried florets were then ground into a fine powder using a freeze grinding machine with a 50 mL container (JXFSTPRP-CLN, Shanghai Jingxin Industrial Development Co., Ltd., Shanghai, China). This procedure was consistent across all samples to minimize pre-analytical variation in metabolite profiles. For pooled sample, 100 mg of dried material per cultivar was combined and further homogenized using the same grinder to generate a pooled sample. Six pooled replicate samples were prepared in this manner and stored at −80 °C until analysis.

For extraction from freeze-dried broccoli samples, 100 mg of freeze-dried broccoli sample powder was added with 1 mL of 70% methanol (*v*/*v*). For fresh broccoli samples, 1 g of fresh sample (equivalent to 100 mg of freeze-dried powder) was placed in a 5 mL pre-cooled (−20 °C) grinding tube, 2 mL of pre-cooled (−20 °C) 100% methanol was immediately added and then the sample was ground into homogenate using a pre-cooled −20 °C freeze grinder. Thereafter, both freeze-dried and fresh samples were then vortexed for 1 min and heated in a water bath at 80 °C for 10 min, followed by ultrasonication for 15 min. Afterward, the supernatant was transferred to a new tube following centrifugation at 8000 rpm for 10 min. The remaining residue was re-extracted by adding 1 mL of 70% methanol, repeating the extraction steps twice for maximum metabolite recovery. Combined the supernatants for vacuum drying and reconstituted in 200 μL of 50% methanol (*v*/*v*). The sample was centrifuged at 13,000 rpm, 4 °C for 15 min, and the supernatant was used for further LC-MS analysis.

### 4.3. UHPLC-Q-TOF MS/MS Analysis Conditions

The chromatographic separation was performed on a Sciex Exion LC system (Foster City, CA, USA) using a Waters ACQUITY UPLC HSS T3 column (50 × 2.1 mm, 1.8 µm) (Waters Corporation, Milford, MA, USA) and a Waters ACQUITY UPLC BEN HILIC column (100 × 2.1 mm, 1.7 μm) (Waters Corporation, Milford, MA, USA). The mobile phase A was water (containing 0.1% formic acid), and the mobile phase B was acetonitrile (containing 0.1% formic acid). The flow rate was set at 0.3 mL/min. The 42 min gradient elution program for the T3 column was described in the following program: 0–2 min, 0% B; 2–3 min, 0–5% B; 3–12 min, 5–10% B; 12–37 min, 10–95% B; 37–39 min, 95% B; 39–42min, 95–5% B. The gradient elution program for the HILIC column was set as: 0–1 min, 95% B; 1–14 min, 95–65% B; 14–16 min, 65–40% B; 16–18 min, 40% B; 18–18.1 min, 40–95% B; 18.1–23 min, 95% B. The column temperature was maintained at 40 °C. The autosampler temperature was set at 15 °C, and the injection volume was 5 µL.

All analyses were performed on a Q TOF 5600 mass spectrometer (Sciex, Foster City, CA, USA) with a Turbo V™ ion source operating in positive and negative electrospray ionization (ESI) mode. MS and MS/MS data were collected for each sample using the TOF mass spectrometer-information-dependent acquisition-enhanced product ion (TOF MS-IDA-EPI) acquisition mode. Data acquisition included a TOF-MS high-resolution scan (*m*/*z* 100–1000 Da) followed by IDA acquisition using a variable window setup (the 10 most intense ions that form the peak of each acquisition cycle were chosen for a product ion scan at *m*/*z* 50–1000 Da). The optimized MS parameters were set as follows: ion spray voltage, 5500 V; the turbo spray temperature, 550 °C; curtain gas, 35 psi; nebulizer gas (gas 1), 55 psi; heater gas (gas 2), 55 psi; declustering potential, 80 V; collision energy, 35 eV; collision energy spread, 15 eV. Data was acquired using SCIEX OS 1.5 Software. In addition, an automated calibration delivery system (CDS) was used to automatically tune the MS and MS/MS every five samples, which performed real-time calibration of the instrument’s mass axis, thereby guaranteeing the continuous accuracy and reliability of the acquired data. Pooled QC samples were injected every 6 runs to monitor signal drift.

### 4.4. Establishment of Published Broccoli Component Database

A comprehensive component database for broccoli was established based on a systematic review of its phytochemical literature. Relevant studies were retrieved from Web of Science and PubMed using search terms including the Latin name (*Brassica oleracea* var. *italica*), chemical constituents, and identification methods. Molecular information of compounds from eligible studies—including compound names, molecular formulas, CAS numbers, structural classifications, and source references—was compiled into the database. To ensure accuracy, all chemical structures were cross validated against PubChem or SciFinder. Compounds lacking traceable original references or insufficient identification evidence were excluded to maintain data reliability.

### 4.5. Establishment of Published Glucosinolates and Related Compounds Database

Broccoli is widely recognized as a rich source of health-promoting phytochemicals, particularly GSLs, nitrogen- and sulfur-containing secondary metabolites critical to plant growth and defense mechanisms in cruciferous species. GSLs are classified into aliphatic, aromatic, and indole subtypes based on their amino acid precursors [20]. Although intact GSLs are chemically stable, tissue disruption activates endogenous myrosinase enzymes, triggering their hydrolysis into bioactive breakdown products such as isothiocyanates and nitriles, which exhibit diverse pharmacological properties [47]. To systematically investigate GSLs and their degradation products in broccoli, a specialized database was developed encompassing GSLs, isothiocyanates, and nitriles. This database integrates known GSL structures and their enzymatic degradation pathways reported in the literature [45,46], enabling targeted identification and functional analysis of these compounds.

### 4.6. The Integrated Identification

The metabolites in broccoli were analyzed using LC-MS/MS with T3 and HILIC columns under positive and negative ESI modes. The two-phase identification approach comprised three sequential steps: (1) Non-targeted metabolite screening: HR-MS and HR-MS/MS data were processed using SCIEX OS, MS-DIAL, and GNPS for molecular networking and spectral similarity analysis. (2) Targeted metabolite identification: putative metabolites identified in the non-targeted phase were cross-referenced against the broccoli component database and validated using targeted MS/MS methods. (3) Final validation was achieved through comparison with authenticated standards by LC-MS, ensuring high confidence in metabolite assignments.

#### 4.6.1. Non-Targeted Analysis

SCIEX OS Platform: The analytical module of SCIEX OS was employed for non-targeted discovery, leveraging the NIST2017 reference database (high-resolution MS/MS spectra of >3000 authenticated standards). Initial identifications underwent rigorous manual curation to eliminate (1) background contaminants detected in procedural blanks and (2) spectral mismatches against the reference library (±10 ppm mass error tolerance).

MS-DIAL Pipeline (https://systemsomicslab.github.io/compms/msdial/main.html, accessed on 15 July 2024) [18]: Raw LC-MS data were converted to Analysis Base File (ABF) format and processed through MS-DIA’s data preprocessing pipeline, encompassing peak picking, chromatographic deconvolution, compound annotation via the platform’s integrated validation database (16,481 ESI(+)-MS/MS spectra of reference compounds), and retention time alignment. Curated results were filtered using identical blank subtraction and spectral match criteria as applied in SCIEX OS analyses.

GNPS Molecular Networking (http://gnps.ucsd.edu/, accessed on 12 July and 15 August 2024) [19]: Converted .mzML files (via MSConvert) were transferred via WinSCP to GNPS for molecular network analysis. Spectral library matching was executed through the GNPS “View Mirror Match” function, comparing experimental MS/MS patterns against public repository entries with a cosine similarity threshold > 0.7.

Data Integration and Validation: Metabolite annotations from all three platforms were consolidated into a unified chemical inventory, extracting CAS registry numbers, molecular formulas, and ClassyFire-based classifications. Duplicate entries were resolved through multidimensional filtering (compound name, retention time ± 0.2 min, and intensity ratio consistency within 20%).

#### 4.6.2. Targeted Metabolite Identification

The Targeted Analysis module of SCIEX OS was implemented to systematically validate metabolites detected in non-targeted screenings. Prioritized targets were selected from three custom phytochemical databases (Section 2.2) based on detection gaps identified through cross-platform comparisons. Candidate compounds were subjected to mass accuracy filtering (<5 ppm) with compounds exhibiting poor peak shape or large mass deviations excluded.

A multidimensional verification workflow was then applied:

Orthogonal Validation: Interrogated extracted ion chromatograms (XIC): Parent ion peak selection was corrected, with manual integration performed where necessary.; HR MS: The *Formula Finder* tool was used to confirm elemental composition, and isotopic intensity patterns were cross validated with theoretical distributions. HR MS/MS: Fragment ion spectra were compared against theoretical and reference fragmentation patterns, excluding low-abundance or artifactual data.

Manual Curation: Excluded candidates with co-eluting interferences (S/N < 10:1); Performed peak-driven reintegration for low-abundance analytes; Enforced retention time consistency (±0.15 min across replicates).

Manual inspection was applied throughout the workflow to resolve redundant, ambiguous, or conflicting annotations, ensuring structural plausibility and avoiding duplication. This tiered validation strategy enabled comprehensive inclusion of database-derived metabolites while maintaining stringent false discovery control (FDR < 1%).

#### 4.6.3. Final Validation

The sample preparation was followed by the method previous described by Hirai’s group [47,48]. Briefly, 4mg dry weight of broccoli florets was accurately weighed and transferred into a 2mL tube with a 5mm zirconia bead YTZ-5 (Watson, Co., Ltd., Murotani, Japan). The metabolites were extracted using a proportional volume of 4 mg/mL extraction solvent (80% methanol, 0.1% formic acid, 210 nmol/L 10-camphorsulfonic acid and 8.4 nmol/L lidocaine as internal standards) using a multibead shocker (Shake Master NEO, Bio Medical Science, Tokyo, Japan) at 1000 rpm for 2 min. After the centrifugation, the extracts were diluted to 1 mg/mL using an extraction solvent. Then, 25 μL of the extract was transferred to a 96-well plate, dried, redissolved in 250 μL of ultrapure water, and filtered using MultiScreenHTS384 well (Merck KGaA, Darmstadt, Germany).

Chromatographic separation was carried out on an ACQUITY UPLC HSS T3 column (100 Å, 1.8 µm, 1 mm × 100 mm; Waters, Milford, MA, USA) using a NexeraX2 UHPLC system (Shimadzu, Kyoto, Japan) coupled to an LCMS-8050 triple quadrupole mass spectrometer (Shimadzu). The mobile phase consisted of solvent A (0.1% formic acid in distilled water) and solvent B (0.1% formic acid in acetonitrile), delivered at a flow rate of 0.24 mL/min. The gradient program was as follows: initial 0.1% B for 0.25 min, increased to 9% B in 0.15 min, to 17% B in 0.40 min, 99.9% B in 1.10 min and kept for 0.2 min, and re-equilibration to 0.01% B at 2.11 min, with a total runtime of 2.7 min. Then, 1 μL of the extract solution was injected.

Mass spectrometric detection was performed in both positive and negative ionization modes. The interface voltage was set at +4 kV in positive mode and −3 kV in negative mode. The interface temperature was maintained at 300 °C, the desolvation line (DL) temperature at 250 °C, and the heat block temperature at 400 °C. Nebulizing gas, drying gas, and heating gas were supplied at 3, 10 and 10 L/min.

## 5. Conclusions

This study addressed the long-standing limitations of broccoli metabolomics—restricted metabolite coverage, losses of labile compounds, and dependence on limited databases—by implementing an integrated analytical workflow that combined optimized extraction, orthogonal chromatographic separation, high-resolution MS/MS acquisition, and tiered non-targeted/targeted annotation strategies. In line with our research hypothesis, this approach successfully overcame methodological constraints, substantially expanding the detectable metabolic space of broccoli and enabling the most comprehensive profiling to date.

The findings confirm that a workflow integrating dual-mode LC separation and complementary annotation strategies can reveal hundreds of previously underrepresented metabolites and achieve higher annotation confidence. Beyond broccoli, the approach establishes a scalable and broadly applicable template for high-confidence, wide-coverage metabolomics of cruciferous vegetables and other complex plant matrices. The resulting dataset not only advances understanding of broccoli’s metabolic architecture but can also be directly incorporated into food composition databases, supporting nutritional research, plant breeding, policy-making, and food innovation.

## Figures and Tables

**Table 1:** 114 metabolites were conclusively identified by targeted metabolomics method.

No	RT	Metabolites	Formula	Calculated [M+H]^+^/[M−H]^−^	Observed [M+H]^+^/[M−H]^−^	Mass Error (ppm)	Column and Mode *	Class **
2	1.55	Pyroglutamic acid	C_5_H_7_NO_3_	128.0353	128.0354	0.7	HILIC, NEG	A
4	1.8	2-Aminoadipic acid	C_6_H_11_NO_4_	162.0761	162.0756	−2.8	HILIC, POS	A
9	3.7	*S*-Methyl-cysteine	C_4_H_9_NO_2_S	136.0427	136.0428	1.2	HILIC, POS	A
13	4.18	Leucine	C_6_H_13_NO_2_	132.1019	132.1013	−4.3	HILIC, POS	A
14	4.29	Tryptophane	C_11_H_12_N_2_O_2_	205.0972	205.0969	−1.3	HILIC, POS	A
14	2.64	Tryptophane	C_11_H_12_N_2_O_2_	205.0972	205.0973	0.9	T3, POS	A
15	4.43	5-Methylcytosine hydrochloride	C_5_H_7_N_3_O	126.0662	126.0659	−2	HILIC, POS	A
19	6.33	Phenylalanine	C_9_H_11_NO_2_	166.0863	166.0857	−3.6	HILIC, POS	A
19	6.35	Phenylalanine	C_9_H_11_NO_2_	164.0723	164.0718	0.8	HILIC, NEG	A
20	6.51	Isoleucine	C_6_H_13_NO_2_	130.0874	130.0876	1.9	HILIC, NEG	A
21	6.6	Methionine	C_5_H_11_NO_2_S	148.0438	148.0438	0.4	HILIC, NEG	A
22	6.61	Tyrosine	C_9_H_11_NO_3_	180.0666	180.0667	0.6	HILIC, NEG	A
22	6.74	Tyrosine	C_9_H_11_NO_3_	182.0812	182.0805	−3.7	HILIC, POS	A
23	6.65	*β*-Homovaline	C_6_H_13_NO_2_	132.1019	132.1013	−4.7	HILIC, POS	A
25	6.68	5-Aminovaleric acid	C_5_H_11_NO_2_	116.0717	116.0717	0.2	HILIC, NEG	A
27	6.69	*N*-Acetyl-glutamic acid	C_7_H_11_NO_5_	188.0564	188.0562	−1.3	HILIC, NEG	A
30	6.86	Norvaline	C_5_H_11_NO_2_	116.0717	116.0718	0.8	HILIC, NEG	A
31	6.87	Threonine	C_4_H_9_NO_3_	118.0510	118.0511	0.9	HILIC, NEG	A
35	7.27	2-Aminobutyric acid	C_4_H_9_NO_2_	102.0561	102.0562	1.1	HILIC, NEG	A
36	7.28	*N*-Acetyl-serine	C_5_H_9_NO_4_	146.0459	146.0462	2	HILIC, NEG	A
38	7.39	Aspartic acid	C_4_H_7_NO_4_	132.0302	132.0304	1.6	HILIC, NEG	A
39	7.39	Allo-threonine	C_4_H_9_NO_3_	118.0510	118.0510	0	HILIC, NEG	A
41	7.59	Pyridoxal hydrochrolide	C_8_H_9_NO_3_	168.0655	168.0653	−1.5	HILIC, POS	A
42	7.61	Carnitine HCl	C_7_H_15_NO_3_	162.1125	162.1123	−1	HILIC, POS	A
45	7.64	Homocarnosine	C_10_H_16_N_4_O_3_	241.1295	241.1292	−1.2	HILIC, POS	A
47	7.65	Serine	C_3_H_7_NO_3_	104.0353	104.0354	0.8	HILIC, NEG	A
50	7.67	Glutamate	C_5_H_9_NO_4_	148.0604	148.0599	−3.7	HILIC, POS	A
58	7.97	Asparagine	C_4_H_8_N_2_O_3_	131.0462	131.0463	0.5	HILIC, NEG	A
62	8.14	Glutamine	C_5_H_10_N_2_O_3_	145.0619	145.0618	−0.3	HILIC, NEG	A
62	8.32	Glutamine	C_5_H_10_N_2_O_3_	147.0759	147.0759	−3.5	HILIC, POS	A
65	8.32	Ala-Ala	C_6_H_12_N_2_O_3_	159.0775	159.0774	−0.9	HILIC, NEG	A
68	8.44	Glutathione (reduced form)	C_10_H_17_N_3_O_6_S	306.0765	306.0764	−0.4	HILIC, NEG	A
70	8.47	Citrulline	C_6_H_13_N_3_O_3_	174.0884	174.0888	2.4	HILIC, NEG	A
70	8.6	Citrulline	C_6_H_13_N_3_O_3_	176.1030	176.103	0.4	HILIC, POS	A
71	8.48	Anserine	C_10_H_16_N_4_O_3_	241.1295	241.1297	0.6	HILIC, POS	A
72	8.51	Methionine sulfoxide	C_5_H_11_NO_3_S	164.0387	164.0388	0.6	HILIC, NEG	A
74	8.52	Cystathionine	C_7_H_14_N_2_O_4_S	221.0602	221.0602	0.1	HILIC, NEG	A
77	8.59	Gly-Gly	C_4_H_8_N_2_O_3_	131.0462	131.0462	0.1	HILIC, NEG	A
78	8.67	Urocanic acid	C_6_H_6_N_2_O_2_	137.0357	137.0357	0.4	HILIC, NEG	A
79	8.69	*S*-Adenosyl- homocysteine	C_14_H_20_N_6_O_5_S	385.1289	385.1282	−1.7	HILIC, POS	A
80	8.72	Ornithine monohydrochloride	C_5_H_12_N_2_O_2_	131.0826	131.0827	0.8	HILIC, NEG	A
81	8.75	Histidine	C_6_H_9_N_3_O_2_	156.0768	156.0762	−3.5	HILIC, POS	A
81	8.82	Histidine	C_6_H_9_N_3_O_2_	154.0624	154.0624	1.5	HILIC, NEG	A
90	9.44	3-Methyl- histidine	C_7_H_11_N_3_O_2_	170.0924	170.0919	−2.9	HILIC, POS	A
93	9.54	Pipecolinic acid	C_6_H_11_NO_2_	130.0863	130.0861	−1	HILIC, POS	A
95	9.57	Lysine	C_6_H_14_N_2_O_2_	147.1128	147.1123	−3.7	HILIC, POS	A
96	9.96	Homomethionine	C_6_H_13_NO_2_S	164.0740	164.0732	−4.8	HILIC, POS	A
97	10.65	Saccharopine	C_11_H_20_N_2_O_6_	277.1393	277.1393	−0.4	HILIC, POS	A
99	10.69	2,3-Diaminopropionic acid monohydrochloride	C_3_H_9_CLN_2_O_2_	141.0425	141.0426	0.3	HILIC, POS	A
100	10.93	Glutathione (oxidized form)	C_20_H_32_N_6_O_12_S_2_	611.1447	611.1454	−2	HILIC, NEG	A
100	10.93	Glutathione (oxidized form)	C_20_H_32_N_6_O_12_S_2_	613.1592	613.158	−2	HILIC, POS	A
107	2.14	Proline	C_5_H_9_NO_2_	116.0706	116.0707	0.7	T3, POS	A
108	2.14	1-Amino-1-cyclopentanecarboxylic acid	C_6_H_11_NO_2_	130.0863	130.0867	3.5	T3, POS	A
151	38.51	Argininosuccinic acid disodium salt	C_10_H_18_N_4_O_6_	291.1299	291.13	0.3	T3, POS	A
162	1.14	Succinic acid	C_4_H_6_O_4_	117.0193	117.0194	0.8	HILIC, NEG	B
171	1.26	Malic acid	C_4_H_6_O_5_	133.0143	133.0143	0.7	HILIC, NEG	B
171	2.25	Malic acid	C_4_H_6_O_5_	133.0144	133.0144	1	T3, NEG	B
174	1.44	Glyceric acid	C_3_H_6_O_4_	105.0193	105.0193	−0.4	HILIC, NEG	B
186	2.08	Citric acid, Anhydrous	C_6_H_8_O_7_	191.0199	191.0199	1.1	HILIC, NEG	B
186	2.3	Citric acid, Anhydrous	C_6_H_8_O_7_	191.0201	191.0201	2.1	T3, NEG	B
199	5.14	Quinic acid	C_7_H_12_O_6_	191.0561	191.056	−0.3	HILIC, NEG	B
206	7.6	1-Aminocyclopropane-1-carboxylic acid	C_4_H_7_NO_2_	100.0404	100.0404	0.3	HILIC, NEG	B
209	7.93	Trigonelline hydrochloride	C_7_H_7_NO_2_	138.0550	138.0543	−4.7	HILIC, POS	B
226	2.28	Citramalic acid	C_5_H_8_O_5_	147.0299	147.0298	−0.8	T3, NEG	B
239	4.15	Threonic acid hemicalcium salt	C_4_H_8_O_5_	135.0299	135.0299	0.3	T3, NEG	B
241	4.68	Pimelic acid	C_7_H_12_O_4_	159.0663	159.0664	0.8	T3, NEG	B
270	16.69	Vanillin	C_8_H_8_O_3_	151.0401	151.0402	0.8	T3, NEG	B
276	18.14	Sebacic acid	C_10_H_18_O_4_	201.1132	201.1132	0.1	T3, NEG	B
368	36.67	Adipic acid	C_6_H_10_O_4_	147.0653	147.0653	0.8	T3, POS	B
376	1.35	Indol-3-ylmethyl-glucosinolate	C_16_H_20_N_2_O_9_S_2_	447.0537	447.0541	0.8	HILIC, NEG	C
376	19.77	Indol-3-ylmethyl-glucosinolate	C_16_H_20_N_2_O_9_S_2_	447.0537	447.0553	3.5	T3, NEG	C
393	3.39	But-3-enylglucosinolate	C_11_H_19_NO_9_S_2_	372.0428	372.043	0.4	HILIC, NEG	C
444	7.89	Sulforaphane	C_6_H_11_NOS_2_	178.0355	178.0354	−0.7	T3, POS	C
477	20.85	Gluconasturtiin	C_15_H_21_NO_9_S_2_	422.0585	422.0587	0.5	T3, NEG	C
511	8.88	Chlorogenic acid Hemihydrate	C_16_H_18_O_9_	353.0878	353.0881	1	T3, NEG	D
521	1.87	4-Pyridoxate	C_8_H_9_NO_4_	182.0459	182.046	0.9	HILIC, NEG	D
529	4.44	Kynurenic acid	C_10_H_7_NO_3_	188.0353	188.0351	−1.1	HILIC, NEG	D
529	4.48	Kynurenic acid	C_10_H_7_NO_3_	190.0499	190.0495	−2	HILIC, POS	D
529	15.82	Kynurenic acid	C_10_H_7_NO_3_	188.0353	188.0355	0.7	T3, NEG	D
534	5.14	Luteolin-3′,7-di-*O*-glucoside	C_27_H_30_O_16_	609.1461	609.146	−0.2	HILIC, NEG	D
547	8.08	2′,6′-Dihydroxy-4-methoxychalcone-4′-*O*-neohesperidoside	C_28_H_34_O_14_	593.1876	593.1874	−0.4	HILIC, NEG	D
549	8.27	3-Hydroxyanthranilic acid	C_7_H_7_NO_3_	154.0499	154.0498	−0.5	HILIC, POS	D
549	4.04	3-Hydroxyanthranilic acid	C_7_H_7_NO_3_	154.0499	154.0499	0	T3, POS	D
555	8.94	Shikimic acid	C_7_H_10_O_5_	175.0601	175.0602	0.7	HILIC, POS	D
576	3.22	Esculin sesquihydrate	C_15_H_16_O_9_	341.0867	341.0869	−0.1	T3, POS	D
600	6.99	cis or trans-4-Hydroxy-3-methoxycinnamic acid_Ferulic acid	C_10_H_10_O_4_	195.0652	195.0658	3.3	T3, POS	D
606	7.36	Aureusidin	C_15_H_10_O_6_	287.0550	287.055	0	T3, POS	D
611	8.29	Glucopyranosyl sinapate	C_17_H_22_O_10_	387.1286	387.1287	0.2	T3, POS	D
611	8.32	Glucopyranosyl sinapate	C_17_H_22_O_10_	385.1140	385.1142	0.5	T3, NEG	D
629	12.3	Quercitrin	C_21_H_20_O_11_	449.1078	449.1082	0.8	T3, POS	D
665	15.63	5-Hydroxyindole-3-acetate	C_10_H_9_NO_3_	190.0510	190.051	0.3	T3, NEG	D
665	15.63	5-Hydroxyindole-3-acetate	C_10_H_9_NO_3_	192.0655	192.0656	0.2	T3, POS	D
696	17.18	Kaempferol-3-rhamnoside-4″-rhamnoside,-7-rhamnoside	C_33_H_40_O_18_	723.2142	723.2168	3.6	T3, NEG	D
717	18.33	Salicylic acid	C_7_H_6_O_3_	137.0244	137.0249	3.8	T3, NEG	D
737	20.62	Sissotrin	C_22_H_22_O_10_	447.1286	447.1284	−0.3	T3, POS	D
790	1.67	Nicotinamide	C_6_H_6_N_2_O	123.0547	123.0547	−4.9	HILIC, POS	E
792	2.13	Riboflavin	C_17_H_20_N_4_O_6_	377.1456	377.1458	0.6	HILIC, POS	E
792	10.74	Riboflavin	C_17_H_20_N_4_O_6_	377.1456	377.1459	0.9	T3, POS	E
793	3.34	Diethanolamine	C_4_H_11_NO_2_	106.0863	106.0859	−3.6	HILIC, POS	E
800	2.13	Pyridoxamine dihydrochloride	C_8_H_12_N_2_O_2_	167.0826	167.082	−3.4	T3, NEG	E
802	2.13	*N*-Acetyl putrescine hydrochloride	C_6_H_14_N_2_O	131.1179	131.1185	4.9	T3, POS	E
810	2.4	Pyridoxine	C_8_H_11_NO_3_	170.0812	170.081	−0.8	T3, POS	E
824	21.29	Indole-3-carboxyaldehyde	C_9_H_7_NO	146.0600	146.0601	0.3	T3, POS	E
831	1.44	Uridine	C_9_H_12_N_2_O_6_	243.0623	243.0623	0.2	HILIC, NEG	F
833	2.07	5′-Deoxy-5′-Methylthioadenosine	C_11_H_15_N_5_O_3_S	298.0968	298.0966	−0.9	HILIC, POS	F
833	2.2	5′-Deoxy-5′-Methylthioadenosine	C_11_H_15_N_5_O_3_S	298.0968	298.098	4	T3, POS	F
835	2.21	Guanosine	C_10_H_13_N_5_O_5_	284.0989	284.0987	−0.7	HILIC, POS	F
836	2.36	Adenosine-3′,5′-cyclicmonophosphate	C_10_H_12_N_5_O_6_P	330.0598	330.0595	−0.8	HILIC, POS	F
837	2.42	2′-Deoxyguanosine monohydrate	C_10_H_13_N_5_O_4_	268.1040	268.1038	−0.7	HILIC, POS	F
838	3.71	Cytidine	C_9_H_13_N_3_O_5_	244.0926	244.0926	−0.8	HILIC, POS	F
842	6.61	Inosine	C_10_H_12_N_4_O_5_	267.0735	267.0728	−2.7	HILIC, NEG	F
845	7.12	Uridine-5′-monophosphate	C_9_H_13_N_2_O_9_P	323.0286	323.029	1.2	HILIC, NEG	F
845	2.48	Uridine-5′-monophosphate	C_9_H_13_N_2_O_9_P	323.0286	323.0284	−0.5	T3, NEG	F
846	7.59	*β*-Nicotinamide mononucleotide	C_11_H_15_N_2_O_8_P	335.0639	335.0634	−1.5	HILIC, POS	F
852	8.08	2′-Deoxyadenosine monohydrate	C_10_H_13_N_5_O_3_	250.0946	250.0943	−1	HILIC, NEG	F
853	8.12	Adenosine	C_10_H_13_N_5_O_4_	266.0895	266.0886		HILIC, NEG	F
855	8.16	Thymidine	C_10_H_14_N_2_O_5_	243.0975	243.0974	−0.6	HILIC, POS	F
859	8.74	2′-Deoxycytidine	C_9_H_13_N_3_O_4_	226.0833	226.0834	0.2	HILIC, NEG	F
867	2.25	Zeatin	C_10_H_13_N_5_O	218.1047	218.1044	−1.4	T3, NEG	F
870	5.35	Zeatin-9-glucoside	C_16_H_23_N_5_O_6_	380.1576	380.1574	−0.5	T3, NEG	F
871	5.89	Adenine	C_5_H_5_N_5_	136.0618	136.0616	−0.9	T3, POS	F
876	10.8	Inosine-5′-monophosphate	C_10_H_13_N_4_O_8_P	349.0544	349.0528	−4.5	T3, POS	F
888	2.88	Sucrose	C_12_H_22_O_11_	341.1089	341.1088	−0.5	HILIC, NEG	G
890	4.37	Cellobiose	C_12_H_22_O_11_	341.1089	341.1087	−0.8	HILIC, NEG	G
891	5.05	Glucoheptose	C_7_H_14_O_7_	209.0667	209.0669	1	HILIC, NEG	G
891	2.22	Glucoheptose	C_7_H_14_O_7_	209.0667	209.0665	−0.7	T3, NEG	G
896	5.19	1-Kestose	C_18_H_32_O_16_	503.1618	503.1618	0.1	HILIC, NEG	G
898	5.36	Melibiose	C_12_H_22_O_11_	341.1089	341.1088	−0.5	HILIC, NEG	G
900	6.76	*α*-Lactose monohydrate	C_12_H_22_O_11_	341.1089	341.1087	−0.8	HILIC, NEG	G
903	7.64	*N*-Acetyl-glucosamine	C_8_H_15_NO_6_	222.0972	222.0972	−0.1	HILIC, POS	G
906	8.02	Raffinose pentahydrate	C_18_H_32_O_16_	503.1618	503.1618	0.1	HILIC, NEG	G

*: Compounds are listed in multiple rows if detected under different chromatographic or ionization conditions. ** A: Amino acids and derivatives; B: Carboxylic acids and derivatives; C: GSL and derivatives; D: Phenolics; E: Alkaloids and related compounds; F: Nucleosides, and analogs; G: Sugars.

**Table 2:** Broccoli florets from 6 cultivars were collected in December 2022 and January 2023.

No.	Cultivar	Provider
Q276	D2206	Wuhan jiutouniao seedling Co., Ltd., Wuhan, China
Q121	Zheqing 227	Zhejiang Academy of Agricultural Sciences, Hangzhou, China
Q287	Zhongqing 15	Institute of vegetables and flowers, Chinese academy of agricultural Sciences, Beijing, China
Q175	W5	Wenzhou Academy of Agricultural Sciences, Wenzhou, China
Q258	B2043	Henan Oulande Seed Industry Co., Ltd., Zhengzhou, China
Q134	Xilanhua 75	Fujian ZhuBo Agriculture Science & Technology Co., Ltd., Ningde, China

## Data Availability

The original contributions presented in this study are included in the article. Further inquiries can be directed to the corresponding authors.

## Supplementary Materials

The following supporting information can be downloaded at: https://www.mdpi.com/article/10.3390/plants14203223/s1, Table S1: Published broccoli component database; Table S2: Published glucosinolates and related compounds; Table S3: Possible metabolites in plants; Table S4: The metabolites in broccoli floret.

## Abbreviations

ABF	Analysis Base File
CDS	Calibration delivery system
EPI	Enhanced Product Ion
ESI	Electrospray ionization
FDR	False Discovery Control
GNPS	Global Natural Products Social Molecular Networking
GSLs	Glucosinolates
HILIC	Hydrophilic interaction
HR-MS/MS	High-resolution tandem mass spectrometry
IDA	Information Dependent Acquisition
LC-HRMS	Liquid Chromatography High Resolution Mass Spectrometry
LC-MS	Liquid chromatography Mass spectrometry
MRM	Multiple Reaction Monitoring
TOF MS	Time of Flight Mass Spectrometry
UHPLC	Ultra High Performance Liquid Chromatography
UHPLC-QTOF MS/MS	Ultra High Performance Liquid Chromatography Quadrupole Time of Flight Tandem Mass Spectrometry
XIC	Extracted Ion Chromatograms
